# Sugammadex Safely Reduces Total Intubation Time in the Intensive Care Unit Following Coronary Artery Bypass Grafting (CABG) at a Real-World Community Hospital

**DOI:** 10.3390/jcm14051660

**Published:** 2025-02-28

**Authors:** Kimberly Lam, Julia Jackson, Chelsey Bourgeois, Elina Delgado, Melissa A. Burmeister

**Affiliations:** 1 Slidell Memorial Hospital, Slidell, LA 70458, USA; kimberly.lam@ochsner.org (K.L.); julia.jackson@ochsner.org (J.J.); chelsey.bourgeois@ochsner.org (C.B.); 2 Department of Pharmacy Practice, School of Pharmacy, William Carey University, Biloxi, MS 39532, USA; 3 Department of Pharmaceutical Sciences, School of Pharmacy, William Carey University, Biloxi, MS 39532, USA

**Keywords:** sugammadex, coronary artery bypass grafting (CABG), neuromuscular blockade (NMB) reversal, fast-track extubation (FTE), train-of-four (TOF)

## Abstract

**Background/Objectives**: Early extubation is crucial for enhancing recovery from coronary artery bypass grafting (CABG). Residual neuromuscular blockade (NMB) effects can hinder early extubation, potentially leading to reintubation, lung infection, and prolonged post-anesthesia stay. Sugammadex, a modified gamma-cyclodextrin, reverses the non-depolarizing NMB effects of the steroidal muscle relaxants rocuronium and vecuronium. The American Society of Anesthesiologists recommends sugammadex administration when patients display a train-of-four (TOF) ratio of less than 0.9. Previous studies show that sugammadex decreases extubation times, reduces postoperative complications, and enhances patient comfort. **Methods**: This single-center, retrospective cohort study evaluated the efficacy of sugammadex in achieving extubation within six hours of intensive care unit (ICU) arrival post-CABG, defined as fast-track extubation (FTE). **Results**: Here, we report that although the total time of intubation in the ICU following CABG did not drop to the six-hour benchmark, it was substantially reduced by the administration of sugammadex in accordance with an FTE protocol. Furthermore, the risks of adverse events (e.g., anaphylaxis, heart failure) and postoperative complications (e.g., acidemia, hypoxemia, tachypnea) were unaltered. **Conclusions**: The use of sugammadex could, thus, reduce costs associated with prolonged intubation time and related complications without increasing morbidity or mortality.

## 1. Introduction

Coronary artery bypass grafting (CABG) is a highly invasive medical procedure that improves blood flow to the myocardium. CABG is generally recommended when there are high-grade blockages in any of the major coronary arteries and/or if percutaneous coronary intervention has failed [1]. CABG is one of the most commonly performed cardiac surgeries worldwide, accounting for ~400,000 procedures annually. The postoperative complications of CABG include stroke, infection, renal failure, atrial fibrillation, and death. With the exception of atrial fibrillation, fewer than 5% of these potentially serious complications occur. In contrast, atrial fibrillation occurs at a substantially higher rate of 20–50% and typically develops within the first five days following CABG [2]. Geriatric age, female sex, nonwhite race, obesity, chronic kidney disease, chronic obstructive pulmonary disease (COPD), and smoking are risk factors that contribute to the development of postoperative complications in CABG partly due to reduced renal clearance, impaired respiratory mechanics, and delayed extubation [3,4,5,6,7,8,9,10,11,12]. Although concerns regarding premature extubation following cardiac procedures were initially touted as reasons to maintain prolonged intubation, results showed no increase in morbidity and mortality, including in high-risk patients [13,14,15,16]. Indeed, increasing evidence indicates that prolonged intubation time increases the risk for experiencing post-surgery complications [17]. Given the mounting evidence in support of early extubation, the Society of Thoracic Surgeons (STS) adopted extubation within six hours after surgery as a quality-of-care benchmark [18].

Neuromuscular blockade (NMB) during surgery involves the use of neuromuscular blocking agents (NMBAs) that cause temporary muscle paralysis. NMBAs are typically administered during anesthesia to facilitate intubation, prevent movement, and optimize mechanical ventilation during surgery. The extent of NMB is routinely assessed by train-of-four (TOF) testing, which employs a peripheral nerve stimulator to measure muscle response (i.e., twitch) after a “train” of four impulses (2 hertz each) is delivered 0.5 s apart [19]. The presence of a fourth twitch (TOF4) indicates 0–5% paralysis; a third twitch (TOF3), 65–75% paralysis; a second twitch (TOF2), 85% paralysis; a first twitch (TOF1), 95% paralysis; and zero twitches, 100% paralysis. Fade, a key characteristic of non-depolarizing (vs. depolarizing) muscle relaxants, is present if the first twitch is evaluated as being stronger than subsequent twitches. The TOF ratio (TOFR) is defined as the ratio of the fourth muscle twitch (i.e., magnitude of muscle twitch expressed as percent normal response) of the TOF sequence to the first one. A lower TOFR indicates a greater degree of paralysis. Adequate recovery from NMB is the return of the TOFR to 0.9 or greater, as this level indicates restoration of the functional integrity of airway muscles involved in airway protection. A TOFR of less than 0.9 signifies residual NMB that requires reversal. The primary goal of the TOF assessment is to ensure that only the minimum amount of a NMBA is administered because, when NMBAs are not fully removed or adequately antagonized systemically, residual paralytic effects can hinder early extubation, potentially leading to upper airway obstruction, reintubation, atelectasis, pneumonia, and/or prolonged stay in the post-anesthesia care unit (PACU) [17].

Approved by the Food and Drug Administration in 2015, sugammadex (Bridion^®^, Merck, Rahway, NJ, USA) selectively reverses the non-depolarizing neuromuscular blocking effects of the routinely used steroidal NMBAs rocuronium and vecuronium [20,21,22,23,24,25,26]. As a modified gamma-cyclodextrin, sugammadex works differently than mainstay NMB reversal agents, such as neostigmine (an acetylcholinesterase inhibitor) and atropine (a competitive muscarinic receptor antagonist). Specifically, sugammadex rapidly and completely reverses rocuronium and vecuronium by chelating the free molecules to form a stable complex, trapping the molecules and reducing their plasma levels, whereby they move from tissue to plasma and nicotinic receptor occupancy at the neuromuscular junction is reduced [26]. There are multiple benefits to choosing sugammadex over traditional NMB reversal agents [27]. Namely, sugammadex is generally associated with a reduced incidence of residual NMB; fewer side effects (e.g., pain, bradycardia, postoperative nausea and vomiting); and faster time to recovery (i.e., achieving a TOFR of 0.9 or greater) compared to neostigmine [28].

Sugammadex can be administered starting at TOF1 [29,30]. Institutional fast-track extubation (FTE) protocols that entail the administration of sugammadex are utilized to expedite patient transfer from the ICU, including following cardiac surgery. Per the STS’s recommendation, extubation following cardiac surgery within six hours indicates a successful FTE protocol [31]. Preliminary data suggest that sugammadex may decrease intubation duration, reduce postoperative complications, and enhance patient comfort following CABG. A randomized, placebo-controlled trial that enrolled 90 participants who were administered sugammadex in accordance with an FTE protocol or placebo reported a decreased time to extubation in sugammadex-treated patients by approximately one hour [32]. No serious adverse events in either the sugammadex- or placebo-treated group were reported, nor were there any benefits to patient outcomes or intensive care unit (ICU) length of stay (LOS) [32]. A retrospective pilot study performed at a tertiary-care hospital compared the number of cardiac surgical patients who could be successfully extubated within six hours of surgery when sugammadex had been administered vs. when it had not been given [1]. Findings indicated that treatment with sugammadex resulted in more CABG patients meeting the STS FTE benchmark extubation criterion post-surgery [1]. One institution that has successfully designed and implemented an effective FTE protocol in cardiac surgical patients is Duke University Hospital (Durham, NC, USA) [33]. In an effort to achieve the STS benchmark as well as reduce the incidence of mortality, our institution has recently adopted Duke University Hospital’s FTE protocol in patients undergoing CABG. Here, the safety and efficacy of sugammadex to reduce total ICU intubation time upon arrival to the ICU post-CABG without increasing risk of adverse effects, postoperative complications, ICU LOS, or hospital LOS were assessed, including after controlling for potential confounding variables that are associated with an increased risk of surgery-related complications, morbidity, and mortality.

## 2. Materials and Methods

This single-center, retrospective cohort study was approved by the Institutional Review Board at Ochsner Health System (New Orleans, LA, USA). Patients who had undergone a CABG procedure between 1 December 2022 and 31 May 2023 were identified using institutional electronic medical records. In June 2023, SMH implemented Duke University Hospital’s FTE protocol, which entailed the administration of sugammadex [33]. CABG patients between 1 June 2023 and 31 December 2023 were also identified. Pre-protocol patients who did not receive sugammadex were compared to post-protocol patients who did. Patients were included if they were 18 years of age and older; received rocuronium (Zemuron^®^, Hospira, Lake Forest, IL, USA) or vecuronium (Norcuron^®^, Eugia, East Windsor, NJ, USA) during the CABG procedure; had an estimated glomerular filtration rate (eGFR) of 30 mL/min/1.73 m^2^ or greater; and displayed a normal core body temperature of 36 °C. Patients were excluded if they had an eGFR of less than 30 mL/min/1.73 m^2^; contraindication or hypersensitivity to sugammadex; postoperative bleeding (i.e., chest tube output greater than 100 mL/h); both COPD and pulmonary hypertension; and/or previously experienced or were at risk for hemodynamic instability beyond expected postoperative course. The primary outcome was the total time of intubation in the ICU, including the percentage of patients who were intubated for longer than twelve hours. The primary safety outcomes were the following adverse reactions: anaphylaxis, acute kidney injury (AKI), new-onset dysrhythmia, and postoperative heart failure (HF). Secondary outcomes were arterial blood gas (ABG) pH, arterial oxygen saturation (SaO_2_), respiratory rate (RR), ICU LOS post-CABG procedure, and total hospital LOS.

Statistical analyses were performed using GraphPad Prism (v. 8.3.1, Boston, MA, USA) and IBM SPSS Statistical (v. 29.0.2.0, Armonk, NY, USA) software. All patients who met the inclusion criteria were included in the analyses. For categorical variables, Fisher’s exact test (for 2 × 2 contingency tables) or chi-square test (for contingency tables larger than 2 × 2) was performed to determine differences between groups. For continuous variables, a student’s *t*-test was performed; data are presented as mean ± standard error of the mean (SEM). Standard deviation (SD) is also provided for all endpoint data. One-way analysis of variance (1W-ANOVA) linear regression analysis was first performed to determine the main effect of sugammadex administration on ICU intubation time. One-way analysis of covariance (1W-ANCOVA) was then performed to determine the covariate effects of geriatric age (i.e., age category), female sex (i.e., sex category), nonwhite race (i.e., race category), and obesity (i.e., body mass index (BMI) category) on total ICU intubation time, as these covariates could potentially impact study outcomes (i.e., contribute to the development of postoperative complications) and confound data interpretation. Effect size, calculated as Cohen’s d, was determined for all *t*-test and linear regression analyses. Effect size was interpreted as very small (0.00–0.19); small (0.20–0.49); medium (0.50–0.79); and large (≥0.80). Statistical power for regression analysis was calculated using the following parameters: power = 0.80, effect size = 0.15. Statistical significance was set to *p* < 0.05.

## 3. Results

**Figure 1:**
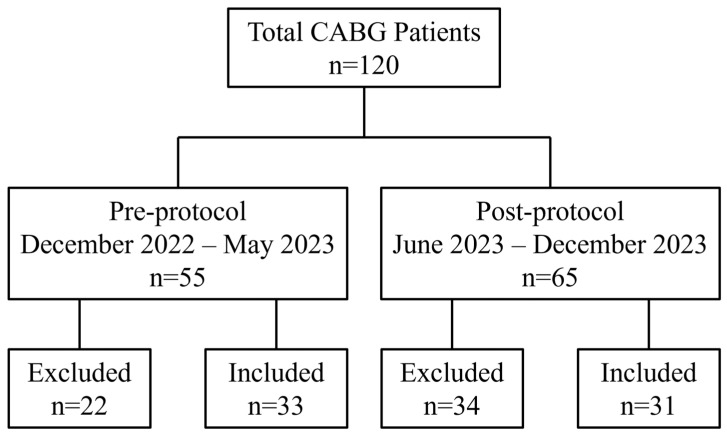
Study enrollment data. A total of *n* = 64 patients were included in the study, with *n* = 33 in the pre-protocol group and *n* = 31 in the post-protocol group.

A total of 120 CABG patients were reviewed (Figure 1). There were 55 and 65 patients in the pre- and post-protocol groups, respectively. Twenty-two pre-protocol and thirty-four post-protocol patients were excluded from the analyses based on the exclusion criteria. Therefore, a total of 64 patients were included in the study, with 33 in the pre-protocol group and 31 in the post-protocol group. The preoperative variables of age (including consideration of age ≥65), sex, race, BMI (including consideration of obese BMI), and eGFR (including consideration of eGFR ≥ 60 mL/min/1.73 m^2^) are presented in Table 1. Intraoperative variables of CABG procedure duration, number of coronary vessels grafted, amounts of each NMBA administered, and amount of sugammadex administered in the post-protocol group are also presented in Table 1. There were no significant differences observed between any of the preoperative variables (Table 1). Regarding intraoperative variables, the duration of the CABG procedure was similar between groups (Table 1). The number of coronary vessels grafted in the pre-protocol group ranged from one to five; this number ranged from one to six in the post-protocol group, with no patients undergoing a two-vessel graft (Table 1). Regardless of study group, the majority of patients underwent a four-vessel graft (Table 1). The average amount of NMBA administered did not differ between the pre- and post-protocol groups, regardless of whether rocuronium or vecuronium was used (Table 1).

**Figure 2:**
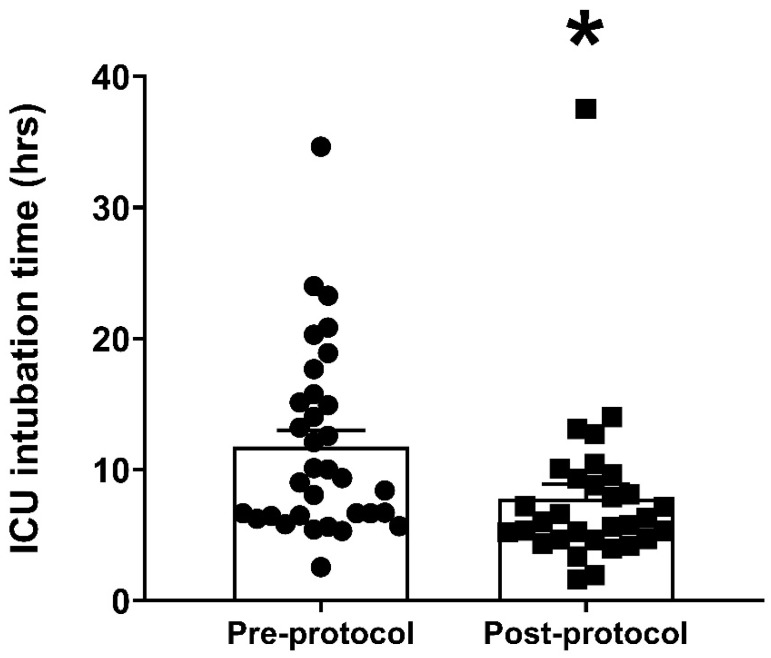
Primary endpoint data. Data represent the total ICU intubation time in patients not receiving sugammadex (pre-protocol) vs. patients receiving sugammadex (post-protocol). Averaged data represent mean ± SEM. * *p* < 0.05 vs. pre-protocol.

The primary outcome of total ICU intubation time was significantly lower in the post-protocol vs. pre-protocol group (7.78 ± 1.13 vs. 11.78 ± 1.23 h, respectively, *p* = 0.020, Cohen’s d = 0.596, effect size: medium), which equated to a 34% reduction in time (Figure 2, Table 2). A substantially lower percentage of post-protocol participants displayed a total ICU incubation time that was longer than twelve hours compared to pre-protocol participants (12.90 vs. 42.42%, respectively) (*p* = 0.012) (Table 2). Primary safety outcomes are also presented in Table 2. A large percentage of patients did not experience any adverse events regardless of study group (pre-protocol: 66.67%; post-protocol: 77.42%) (Table 2). There were no cases of anaphylaxis across groups, and the incidence of new-onset dysrhythmia as well as postoperative HF were similar between the pre- and post-protocol groups (Table 2). Although a larger percentage of pre-protocol participants experienced AKI compared to the post-protocol group (18.18 vs. 6.45%, respectively), the overall incidence of adverse events did not differ between groups. There were no statistically significant differences observed between any of the safety outcomes (Table 2). The safety outcomes assessed were post-surgery respiratory status, as reflected by arterial pH (ABG pH) and oxygenation levels (SaO_2_) as well as respiratory rate (RR), ICU LOS, and total hospital LOS (Table 2). The corresponding effect sizes ranged from very small to small.

The study did not meet statistical power, as power analysis of linear regression determined that the minimum sample size required to detect an effect was 92. Nevertheless, the assumptions of normal distribution and homogeneity of variance-covariance were met, allowing for parametric linear regression analyses. One-way ANOVA linear regression analysis determined that the observed difference in total ICU intubation time between the pre- and post-protocol groups was significantly (*p* = 0.020) associated with NMB reversal using sugammadex (Table 3). The association between sugammadex use and reduced ICU intubation time remained significant (*p* = 0.034) after adjusting for the covariates of age, sex, race, and BMI (Table 3). Corresponding effect sizes were very small.

## 4. Discussion

Concerns that premature extubation following cardiac surgery increases the risk of adverse events, complications, as well as morbidity and mortality have largely proven to be unfounded. Indeed, the STS has adopted extubation within six hours after surgery as a quality-of-care benchmark [18]. As part of this initiative, the NMB reversal agent sugammadex is being more routinely used to selectively and reliably reverse the neuromuscular blocking effects of rocuronium and vecuronium and quickly restore neuromuscular function without increasing adverse events, including in major cardiothoracic surgery and particularly in high-risk patients [16,34,35,36,37]. Here, we report that the use of sugammadex as part of an FTE protocol to reverse the neuromuscular blocking effects of rocuronium and vecuronium following CABG significantly reduces total ICU intubation time by 34% without significantly increasing the risk of adverse effects or complications, including extending ICU LOS and total hospital LOS, in a real-world community hospital adult patient population. The reductions in the average ICU intubation time to well below twelve hours as well as in the percentage of patients intubated for longer than twelve hours becomes especially important because the risk of postoperative complications increases when the intubation time exceeds this length of time [38]. Moreover, the sugammadex-mediated reduction in ICU intubation time persisted when geriatric age, female sex, nonwhite race, and obesity, which are risk factors for increased morbidity and mortality following CABG surgery, were factored as covariates [3,4,5,10,11,12].

The present findings align with a growing body of evidence demonstrating the benefits of early extubation in terms of improved patient outcomes [17,33]. The primary benefits of NMB reversal with sugammadex are related to it being quicker acting and more effective at completely restoring muscle strength than the traditional reversal agent neostigmine. These actions of sugammadex are especially beneficial in surgical patients with concomitant complications that impair breathing; increase the risk of arrhythmia, hemodynamic instability and neuromuscular blockade; reduce renal clearance; and delay extubation, as seen in advanced age, obesity, and COPD [3,4,5,6,7,8,9,10,11,12,39,40,41]. Also, the efficacy of sugammadex is not influenced by the choice of anesthetic, and dose adjustments are not required in elderly patients [42,43,44,45]. As a result, anesthesiologists are more likely to elect for a deeper level of NMB when sugammadex is the reversal agent, which itself is associated with better operative and postoperative conditions in thoracic surgery [35,46,47]. Multiple benefits of NMB reversal with sugammadex in CABG and other cardiothoracic surgeries have been described. Bardia et al. report that NMB reversal with sugammadex is associated with a (albeit small) reduced time to extubation in patients undergoing CABG, aortic valve replacement (AVR), or a combination of CABG and AVR vs. placebo [17]. In a larger study by Greenberg et al., the reversal of rocuronium with sugammadex not only resulted in more patients meeting the STS benchmark extubation criteria following cardiac surgery but also significantly reduced time to extubation compared to patients not receiving sugammadex [1]. In patients undergoing transcatheter AVR via a transapical implantation approach, the reversal of rocuronium-induced NMB with sugammadex is associated with a decreased incidence of postoperative pulmonary complications (PPCs) (e.g., respiratory failure, respiratory infection, bronchospasm, atelectasis, pleural effusion, pneumothorax, aspiration pneumonitis, new-onset hypoxemia) compared to reversal of the non-depolarizing NMBA cisatracurium with neostigmine [48]. Numerous other studies report the superiority of sugammadex over neostigmine in terms of reducing PPCs [36,49,50,51,52,53,54,55,56]. Sugammadex also improves postoperative cognitive outcomes, which could be especially beneficial in elderly cardiac surgical patients in whom the risk of delirium may be increased [57,58]. In patients undergoing AVR via cardiopulmonary bypass, postoperative memory deficit is reverted by sugammadex and is accompanied by an increased expression of anti-inflammatory microglial markers; however, there are no short-term decreases in ICU length of stay and overall hospital stay [57]. Indeed, the superior efficacy of sugammadex has even sparked debate about whether quantitative neuromuscular monitoring should still be required [59].

While the safety profile of sugammadex is generally good, potential risks and limitations have been reported. Adverse events include anaphylaxis, bradycardia, hypotension, bronchospasm, and cardiac arrest; serious adverse events include angioedema, laryngeal edema, pulseless electrical activity, and Kounis syndrome [60,61]. However, the composite incidence of bradycardia, anaphylaxis, bronchospasm, and cardiac arrest in patients who had general, cardiothoracic, or pediatric surgery is not different between neostigmine and sugammadex [62]. Other reported side effects include coughing, limb or body movement, parosmia, and elevated urine levels of *N*-acetyl-glucosaminidase (a marker of renal tubular injury) [63]. There are also reports of increased risk of anaphylaxis and related complications [64,65]. In contrast, risk of anaphylaxis to neostigmine appears negligible [66,67,68]. Neostigmine allows for more broad-spectrum NMB compared to sugammadex, which is limited to rocuronium- and vecuronium-induced NMB, and neostigmine may be a better choice for thoracic surgery patients when the depth of blockade is light or minimal [36]. Another potential drawback to using sugammadex is that the agent can bind to endogenous and pharmaceutical molecules besides steroidal NMBAs (e.g., oral contraceptives), thereby reducing their efficacy as well as resulting in NMB reoccurrence due to the displacement of rocuronium or vecuronium [69,70].

Claims of sugammadex’s superiority over neostigmine could perhaps be debunked by evidence that inappropriate use of neostigmine contributes to increased postoperative residual NMB compared to sugammadex [36]. Initiating NMB with neostigmine at a TOF count of less than 3 or 4; not allowing enough time between neostigmine administration and tracheal extubation; not confirming a TOFR of 0.9 prior to extubation; using high-dose neostigmine; and lack of qualitative or quantitative monitoring are deficiencies in clinical practice that could contribute to a high incidence of postoperative residual NMB [36,52,53,54]. Indeed, several studies report that the rate of PPCs is the same between neostigmine and sugammadex [36,50,71,72]. Ultimately, pharmacovigilance when using any neuromuscular blocking reversal agent is paramount to avoid residual NMB and recurrent paralysis. With sugammadex, the required dose is typically less than but can sometimes be greater than the recommended dose based on TOF testing [23]. Compared to neostigmine, the higher cost of sugammadex could be a barrier to use, although this obstacle could be circumvented with justifications of reduced costs overall related to reduced length of hospitalization [51,73,74,75,76]. However, the lack of improvements in anesthesia time, operating time, and time spent in PACUs may not justify the unrestricted use of sugammadex [36].

Although the STS extubation benchmark was not achieved in sugammadex-treated patients in the present study, it is likely not attributable to a lack of drug efficacy but rather several limitations. Namely, the study is limited by its retrospective design and small sample sizes (i.e., being underpowered). There is a lack in diversity in both study groups, which consisted of patients who were predominantly of geriatric age, male sex, and White race. There were instances of protocol deviation, whereby patients who could have received sugammadex prior to extubation did not. Potential contributors to protocol deviation include not having enough staff members to assist with extubation and/or staff unawareness of the institution’s implementation of the FTE protocol. Moreover, TOF testing was not performed due to a shortage of TOF monitoring devices, with only two machines on-hand for the ICU. Lastly, the lack of generalizability is a significant limitation, as the study was conducted at only a single institution. Despite these challenges, SMH strives to emulate the operational practices set forth by the cardiothoracic ICU team at Duke University Hospital, which has utilized the Define, Measure, Analyze, Improve, and Control quality improvement approach to successfully implement an FTE protocol and achieve the STS benchmark [33]. The team first determined that extubation time in cardiac surgery patients undergoing CABG, valve repair or replacement, ascending aortic aneurysm repair, maze procedure, septal myectomy, and combinations of these procedures was highly variable [33]. This variability was partly attributable to process-, people-, and patient-specific barriers (process-specific: a lack of clarity about eligibility for early extubation, use of sedation, and inadequate pain management; people-specific: a lack of interdisciplinary communication and absence of staff cross-coverage; patient-specific: metabolic or respiratory acidosis, hemodynamic instability, bleeding, and altered mental state). Following a multidisciplinary FTE intervention that incorporated (1) a standardized workflow with prespecified safety parameters built into its design and (2) the influencer change model focused on motivationally changing behavior, there was a significant and sustained improvement in the rate of extubation within six hours of cardiac surgery; reduction in process- and people-specific barriers; and potential increase in cost savings [33].

## 5. Conclusions

Taken together, the present findings corroborate a growing body of evidence indicating that implementing FTE protocols that incorporate sugammadex could significantly improve patient outcomes post-CABG without increasing the risk of adverse events, postoperative complications, morbidity, or mortality, as well as reducing costs associated with prolonged intubation time.

## Figures and Tables

**Table 1:** Preoperative and intraoperative variables.

Variable		Pre-Protocol (*n* = 33)	Post-Protocol (*n* = 31)	*p*-Value
Age, years		69.36 ± 1.80	67.19 ± 2.24	0.450
Age ≥ 65, *n* (%)		25 (75.76%)	19 (61.29%)	0.283
Male sex, *n* (%)		24 (72.73)	20 (64.52)	0.592
White race, *n* (%)		26 (78.79)	29 (93.55)	0.150
Body mass index (BMI), kg/m^2^		30.05 ± 0.96	31.31 ± 1.23	0.422
Obese BMI, *n* (%)		17 (51.52%)	15 (48.39)	>0.999
eGFR, mL/min/1.73 m^2^		56.88 ± 1.17	57.21 ± 1.31	0.850
eGFR ≥ 60 mL/min/1.73 m^2^, *n* (%)		25 (75.76)	25 (80.65)	0.765
Duration of CABG procedure, hours		5.14 ± 0.25	5.72 ± 0.20	0.080
Number of coronary vessels grafted, *n* (%)	1	5 (15.15)	1 (3.23)	0.090
	2	3 (9.09)	0 (0.00)	
	3	7 (21.21)	5 (16.13)	
	4	14 (42.42)	14 (45.16)	
	5	4 (12.12)	10 (32.26)	
	6	0 (0.00)	1 (3.23)	
NMBA amount administered, mg	Rocuronium	68.48 ± 9.10	69.03 ± 7.64	0.964
	Vecuronium	13.09 ± 1.42	10.26 ± 0.97	0.109
Sugammadex amount administered, mg		0.00 (0.00)	184.71 ± 8.60	<0.001 *

* *p* < 0.05.

**Table 2:** Primary, safety, and secondary endpoint data.

Endpoint Data	Pre-Protocol (*n* = 33)	Post-Protocol (*n* = 31)	Cohen’s d	*p*-Value
Primary outcomes				
ICU intubation time, hours (SD)	11.78 ± 1.23 (7.07)	7.78 ± 1.13 (6.31)	0.596	0.020 *
ICU intubation time >12 h, *n* (%)	14 (42.42)	4 (12.90)	0.328	0.012 *
Primary safety outcomes				
Anaphylaxis, *n* (%)	0 (0.00)	0 (0.00)	0.178	0.567
Acute kidney injury, *n* (%)	6 (18.18)	2 (6.45)		
New onset of dysrhythmia, *n* (%)	4 (12.12)	4 (12.90)		
Postoperative heart failure, *n* (%)	1 (3.03)	1 (3.23)		
No adverse events, *n* (%)	22 (66.67)	24 (77.42)		
Secondary outcomes				
ABG pH (SD)	7.37 ± 0.01 (0.05)	7.35 ± 0.01 (0.06)	0.484	0.057
SaO_2_, % (SD)	97.15 ± 0.33 (1.91)	97.10 ± 0.53 (2.94)	0.022	0.929
Respiratory rate, breaths/min (SD)	18.06 ± 0.77 (4.44)	16.90 ± 0.56 (3.13)	0.299	0.236
ICU LOS post-CABG, days (SD)	6.18 ± 0.45 (2.60)	5.71 ± 0.29 (1.62)	0.216	0.390
Total hospital LOS, days (SD)	10.70 ± 1.06 (6.07)	9.84 ± 0.72 (4.00)	0.166	0.510

* *p* < 0.05.

**Table 3:** Statistical analyses of main and covariate effects.

Statistical Test	Covariates	Main Variables		Point Estimates $\bar{X}$	Parameter Estimates		Cohen’s d	*p*-Value
		Independent	Dependent		95% Confidence Interval			
					Lower Bound	Upper Bound		
1-W ANOVA	None	Protocol category	ICU intubation time	4.002	0.644	7.360	0.084	0.020 *
1-W ANCOVA	Age category, sex category, race category, body mass index category	Protocol category	ICU intubation time	3.927	0.316	7.538	0.076	0.034 *

* *p* < 0.05.

## Data Availability

The data presented in this study are available on request from the corresponding author due to them being collected from patient electronic medical records.

## Abbreviations

The following abbreviations are used in this manuscript:

1W-ANOVA	One-way analysis of variance
1W-ANCOVA	One-way analysis of covariance
ABG	Arterial blood gas
AKI	Acute kidney injury
AVR	Aortic valve replacement
BMI	Body mass index
CABG	Coronary artery bypass grafting
COPD	Chronic obstructive pulmonary disease
eGFR	Estimated glomerular filtration rate
FTE	Fast-track extubation
HF	Heart failure
ICU	Intensive care unit
LOS	Length of stay
NMB	Neuromuscular blockade
NMBA	Neuromuscular blocking agent
PACU	Post-anesthesia care unit
PPC	Postoperative pulmonary complication
RR	Respiratory rate
SaO_2_	Arterial oxygen saturation
STS	Society of Thoracic Surgeons
TOF	Train-of-four
TOFR	Train-of-four ratio
